# The Impact of a Stochastic Parameterization Scheme on Climate Sensitivity in EC‐Earth

**DOI:** 10.1029/2019JD030732

**Published:** 2019-12-08

**Authors:** K. Strommen, P. A. G. Watson, T. N. Palmer

**Affiliations:** ^1^ Department of Physics University of Oxford Oxford UK

## Abstract

Stochastic schemes, designed to represent unresolved subgrid‐scale variability, are frequently used in short and medium‐range weather forecasts, where they are found to improve several aspects of the model. In recent years, the impact of stochastic physics has also been found to be beneficial for the model's long‐term climate. In this paper, we demonstrate for the first time that the inclusion of a stochastic physics scheme can notably affect a model's projection of global warming, as well as its historical climatological global temperature. Specifically, we find that when including the “stochastically perturbed parametrization tendencies” (SPPT) scheme in the fully coupled climate model EC‐Earth v3.1, the predicted level of global warming between 1850 and 2100 is reduced by 10% under an RCP8.5 forcing scenario. We link this reduction in climate sensitivity to a change in the cloud feedbacks with SPPT. In particular, the scheme appears to reduce the positive low cloud cover feedback and increase the negative cloud optical feedback. A key role is played by a robust, rapid increase in cloud liquid water with SPPT, which we speculate is due to the scheme's nonlinear interaction with condensation.

## Key Points

- The inclusion of a stochastic scheme reduces climate sensitivity in a general circulation model
- This reduction, of around 10%, is linked to changes in cloud cover and cloud optical depth feedbacks
- Well‐calibrated stochastic schemes may give more accurate global warming projections

## 1 Introduction

Estimating the extent of global warming due to anthropogenic forcing is one of the primary challenges in climate science and, arguably, one of the most pressing problems to address for society as a whole. However, despite significant efforts in model development, estimates from state of the art climate models have remained relatively unchanged since the original Intergovernmental Panel on Climate Change report (Sherwood et al., 2014). The majority of the spread in climate sensitivity across models can be attributed to differences in the response of clouds to an increase in greenhouse gases (Dufresne & Bony, 2008), with low‐level cloud response being particularly crucial (Bony & Dufresne, 2005; Zelinka et al., 2012a). Because the equations of physics that modulate the hydrological cycle and, therefore, cloud cover are known to first order (Palmer, 2016), variations across the models' representation of clouds are due to different choices in how to truncate these equations to a finite resolution. Of particular importance here are the simplified parameterizations used to determine the contribution from the subgrid‐scale physics. Because the subgrid‐scale contribution is not uniquely constrained by the grid‐scale state, there remain considerable amounts of choice involved in implementing these. While parameterizations have become increasingly sophisticated, they are still the dominant source of uncertainty in climate models.

One source of simulation error in subgrid‐scale processes is the fact that a large portion of the variability at these scales is inherently unpredictable (Cohen & Craig, 2006; Davies et al., 2013). In medium‐range and seasonal forecasts using numerical weather prediction models, the use of stochastic schemes has become widespread as a means to sample this variability. Studies have shown that, when properly calibrated, such schemes have a beneficial impact on both the spread and mean state of these forecasts (Berner et al., 2017; Leutbecher et al., 2017; Weisheimer et al., 2011). In recent years, there has also been increasing interest in understanding the impact of these schemes on the long‐term climate of a model. In Palmer (2012), it was argued that introducing stochasticity into climate models may be a key step toward eliminating persistent model biases and reducing uncertainty in climate projections, a view corroborated further by Christensen and Berner (2019). Since then, the insertion of a stochastic component into a climate model has been demonstrated to improve several key processes, including the El Niño–Southern Oscillation (Berner et al., 2018; Christensen et al., 2017a), the Madden Julian Oscillation (Wang & Zhang, 2016), and the representation of the Indian monsoon (Strømmen et al., 2018). Improvements were also found on regime behavior, northern hemispheric blocking patterns, and tropical precipitation (Dawson & Palmer, 2015; Davini et al., 2017; Watson et al., 2017). Most of these studies focused on a particular, multiplicative noise scheme called the “stochastically perturbed parametrization tendencies” (SPPT) scheme (see section 2.2). A more flexible variant of this scheme, dubbed “independent SPPT” (ISPPT) was developed and found to substantially improve the skill of weather forecasts in areas with significant convective activity (Christensen et al., 2017b). In MacLeod et al. (2016), stochasticity was added to the land scheme of the Integrated Forecast System (IFS) and was found to have a positive impact on seasonal predictability, as well as the representation of the 2003 European heat wave.

Strømmen et al. (2019) found that SPPT, the stochastic land scheme, and ISPPT, substantially affected the mean state of the EC‐Earth model by considering an ensemble of simulations with forced sea surface temperatures (SSTs). All three schemes, particularly the two atmospheric schemes, were found to notably change the model's energy budget and thereby surface temperature. In addition, both the vertical distribution of cloud cover and the liquid water content of the clouds were found to change when turning on the stochastic schemes, and these changes were hypothesized to be responsible for the major changes in the energy budget and hydrological cycle.

In this paper we demonstrate, for the first time, that SPPT also substantially affects 21st century global warming in a coupled general circulation model, reducing it by 10%. The scheme also changes the model's mean state, in particular reducing the global mean surface temperature. Both changes are linked to a change in the modeled clouds due to SPPT.

The paper is structured as follows. Section 2 contains information about the data used: We describe the EC‐Earth model, the experiments considered and the SPPT scheme. Statistical methods are also described. In section 3, we document and visualize the results of the paper: We show the change in historical mean temperatures and the reduced climate sensitivity. Section 4 contains our analysis on mean state changes. These are linked, to first order, to rapid changes in the cloud liquid water content (CLWC) of clouds through most of the vertical layers. This has the effect of increasing the optical thickness (and hence albedo) of clouds, reducing incoming solar radiation and thereby cooling the surface. The reduced level of global warming is analyzed in section 5 and is linked to two factors. First, the scheme appears to reduce the low‐level cloud cover feedback by slowing down the trend of reduced low‐level cloud cover seen in the model. This may be because clouds with more water tend to break up less easily when subjected to an increased temperature. Second, the increased cloud water due to SPPT leads to a slight increase in the negative cloud optical feedback. We also discuss the possible impact of potential nonlinearity in global warming feedbacks. Concluding remarks are made in section 6.

## 2 Data and Methods

### 2.1 The EC‐Earth Model

EC‐Earth v3.1 is an Earth system model developed by the international EC‐Earth consortium (Hazeleger et al., 2012). The atmospheric component uses a modified version of the IFS used by the European Centre for Medium‐Range Weather Forecasts (ECMWF). Land surface processes are simulated using the Hydrology Tiled ECMWF Scheme of Surface Exchanges over Land (H‐TESSEL) (Balsamo et al., 2009). The atmosphere is dynamically coupled to the ocean model “Nucleus for European Modelling of the Ocean” (NEMO) model Version 3.6. The coupling in this case is handled with OASIS3 (Valcke, 2013). For all experiments considered, the spectral truncation (i.e., resolution) of the IFS component is T255 (corresponding to roughly 80‐km grid spacing near the equator), and the ocean resolution is 1°.

### 2.2 Description of the Stochastic Scheme SPPT

The SPPT scheme has been used in ECMWF's operational ensemble forecasts since 1998 and is designed to represent forecast uncertainty that arises from the unpredictable subgrid‐scale variability. This is done by perturbing the total net tendency from the physics parametrizations using multiplicative noise:

(1) $$P_{perturbed}=\left(1+\mu r\right)\sum_{i=1}^{6}P_{i},$$

where **P** is the tendency vector (of a given variable) from the *i*th physics parametrization scheme and *r* a random variable. Note that only tendencies for prognostic model variables (winds, temperature, and specific humidity) are perturbed. Diagnostic variables (such as cloud water) are computed as normal using the prognostic variables. The perturbation *r* is a random scaling factor that is constant in the vertical, with the scaling tapered by *μ*∈[0,1], which is smoothly reduced to 0 in the boundary layer and stratosphere, and 1 otherwise. Roughly speaking, *μ*=1 between pressure levels 900 and 100 hPa (see Palmer et al., 2009). Furthermore, *r* follows a Gaussian distribution with mean zero and is smoothly correlated in space and time. The implementation in EC‐Earth follows that in the Integrated Forecasting System as described in Palmer et al. (2009). The total perturbation *r* is generated by summing over three independent spectral patterns with standard deviations [0.52, 0.18, 0.06], spatial correlation lengths [500, 1,000, 2,000 km], and temporal decorrelation scales [6 hr, 3 days, 30 days], respectively. The perturbation *r* is limited to the range −1 to 1 (roughly two standard deviations of the largest‐amplitude pattern), which ensures that the perturbation is not too large: In particular, **P**_*perturbed*_ always has the same sign as the “deterministic tendency” $P=\sum P_{i}$.

Note that even though the perturbations are mean zero, because of the nonlinearity of the underlying equations, the introduction of such perturbations may have systematic effects on the model state, such as changing the amount of energy in the system. Since the scheme is actively trying to represent unresolved subgrid‐scale variability and this variability has a nonzero amount of energy associated to it, such an injection of energy is plausibly beneficial to the model. In fact, the excessive dissipation of small‐scale energy in climate models has been used as motivation for the development of so‐called “backscatter schemes,” which explicitly reinsert estimates of this dissipated energy back into the large scale (Berner et al., 2009; Tennant et al., 2011).

Detailed information about the physics parameterizations and computation of diagnostic variables in the IFS can be found in ECMWF (2017).

### 2.3 The SPHINX Experiments

This study was based on a set of coupled simulations of the EC‐Earth model carried out as part of the “Climate SPHINX Project,” hereby referred to simply as SPHINX: “Stochastic Physics and HIgh resolution eXperiments” (Davini et al., 2017). Each simulation spans the years 1850–2100, with historical forcings before 2010 and RCP8.5 forcings (Riahi et al., 2011) from then on. Three simulations were “deterministic” (i.e., without SPPT), and three were run with SPPT.

In order to generate suitable initial conditions for the two sets of experiment, a spin‐up phase was carried out, in which the model is run with constant 1850s forcings for 100 years. Two separate such spin‐ups were performed: one with SPPT turned on and one without. The former produced the initial ocean state used for the stochastic simulations and the latter the state used for the deterministic simulations. In each case, tiny perturbations to this state are added to generate three distinct sets of initial conditions, which are then used to generate the ensemble. In particular, the stochastic and deterministic simulations are starting from entirely distinct initial conditions.

Further details of the model configuration can be found in Davini et al. (2017). Of particular note is the introduction, in the stochastic version of the model, of a “humidity fix.” It was found that the SPPT scheme does not conserve water, leading to an unphysical drying of the atmosphere. The “fix” computes, at each time step, global mean precipitation, and evaporation, and reinserts the amount of humidity required to bring these into balance. This humidity is reinserted with spatial weighting favoring regions where the imbalance is large. The possible impact of this fix on the results are discussed by reference to a control experiment described in the next section.

### 2.4 The “FastSPHINX” Experiments

As emphasized in the previous section, the deterministic and stochastic ensembles originate from two distinct spin‐ups. As we will see, these two spin‐ups do not lead to equivalent climates: In particular, the stochastic simulations are starting from a different mean state than the deterministic simulations. Because of this, it is not possible to identify the fast changes in the model caused by turning on SPPT from the SPHINX simulations alone. This makes the determination of any root cause of mean state changes very difficult, as most of the key changes will already have taken place during the spin‐up phase. As a concrete example, we will show that SPPT systematically affects both surface temperatures and clouds, and it is not possible to determine from the SPHINX experiments if the cloud changes were forcings of, or responses to, the temperature changes, as both changes are already in place at the start of the simulations.

Therefore, we additionally performed two new ensemble experiments, consisting of 10 pairs of new simulations using the exact same model as used in SPHINX. Each pair consists of a deterministic and stochastic simulation starting from the exact same initial condition. Crucially, the underlying model climate used to generate the initial conditions are the same for both, namely, the spun up state of the deterministic SPHINX simulations. As a result, in each pair, the SPPT simulation immediately diverges from the deterministic counterpart due to the turning on of the stochastic scheme, and therefore, these experiments allow us to identify the rapid response in the model to SPPT. In particular, we will use these experiments to address the question of causality raised above. These are dubbed the “FastSPHINX” experiments.

As the base state used to generate initial conditions is the spun up deterministic model, the experiments all start from a climate generated with constant 1850s forcing. Small perturbations to this state are allowed to evolve for 1 year with this fixed forcing to produce 10 distinct initial conditions. In order to account for any potential seasonal influence on the signal, five of these initial conditions are set to have start dates on 1 February and five on the 1 August. Each deterministic/stochastic pair is then run for 6 months, again with fixed 1850s forcing; the final data analyzed are composed of these final 6 months. For the results shown, no meaningful seasonal influence on the signal was found, so we will typically present data using all start dates with no further comment. The reader can therefore simply think of the FastSPHINX experiments as two 10‐member ensembles of EC‐Earth, starting in 1850 and running for 6 months, all starting from the same underlying climate. For clarity, Table 1 provides a summary of both the SPHINX and FastSPHINX experimental configurations.

**Table 1:** Description of Deterministic (Det) and Stochastic (Stoch) Model Simulations Considered

	Spin‐up settings	Start date	Duration	Forcing scenario	Number of members
SPHINX ‐ Det	100 years; no SPPT	Jan 1850	250 years	Historical+RCP8.5	3
SPHINX ‐ Stoch	100 years; with SPPT	Jan 1850	250 years	Historical+RCP8.5	3
FastSPHINX ‐ Det	1 year; no SPPT	Feb/Aug 1850	6 months	Fixed 1850s	10
FastSPHINX ‐ Stoch	1 year; no SPPT	Feb/Aug 1850	6 months	Fixed 1850s	10

To test the robustness of the results, additional experiments were also performed. First, a similar ensemble with forced SSTs was carried out, and second, an experiment to test the potential impact of the “humidity fix” was also carried out by running a simulation with SPPT turned on but the “fix” turned off.

In all simulations, the tuning parameters of EC‐Earth are identical for both deterministic and stochastic simulations. Consequently, the *only* difference between the deterministic and stochastic model is the turning on of SPPT in the latter, as well as the turning on of the “humidity fix” mentioned previously.

### 2.5 Statistical Methods

When considering the SPHINX simulations, we do not apply formal statistical tests, due to the small sample size of 3. Instead, we typically allow the data to speak for itself by plotting all ensemble members together. For the large changes observed, all three stochastic ensemble members will usually be greater/smaller than all three deterministic counterparts. For long time series, such a clear divergence over multiple decades would be

highly unlikely if the distributions drawn from are in fact the same. In the absence of further ensemble members, a clear such divergence over time will be taken as our criteria for significance when considering SPHINX data.

For the FastSPHINX experiments, statistical significance is calculated using a two‐tailed *T* test, with no assumption of equal variance across the samples. When means are estimated, error bars (e.g., indicated with shading on time series plots) are defined using the standard error. The standard error is computed as the standard deviation across the sample divided by the square root of the number of samples (i.e., the square root of 10 in our case). When considering differences, we always pair up the corresponding deterministic/stochastic simulations that started from the same initial condition.

## 3 Results

**Figure 1:**
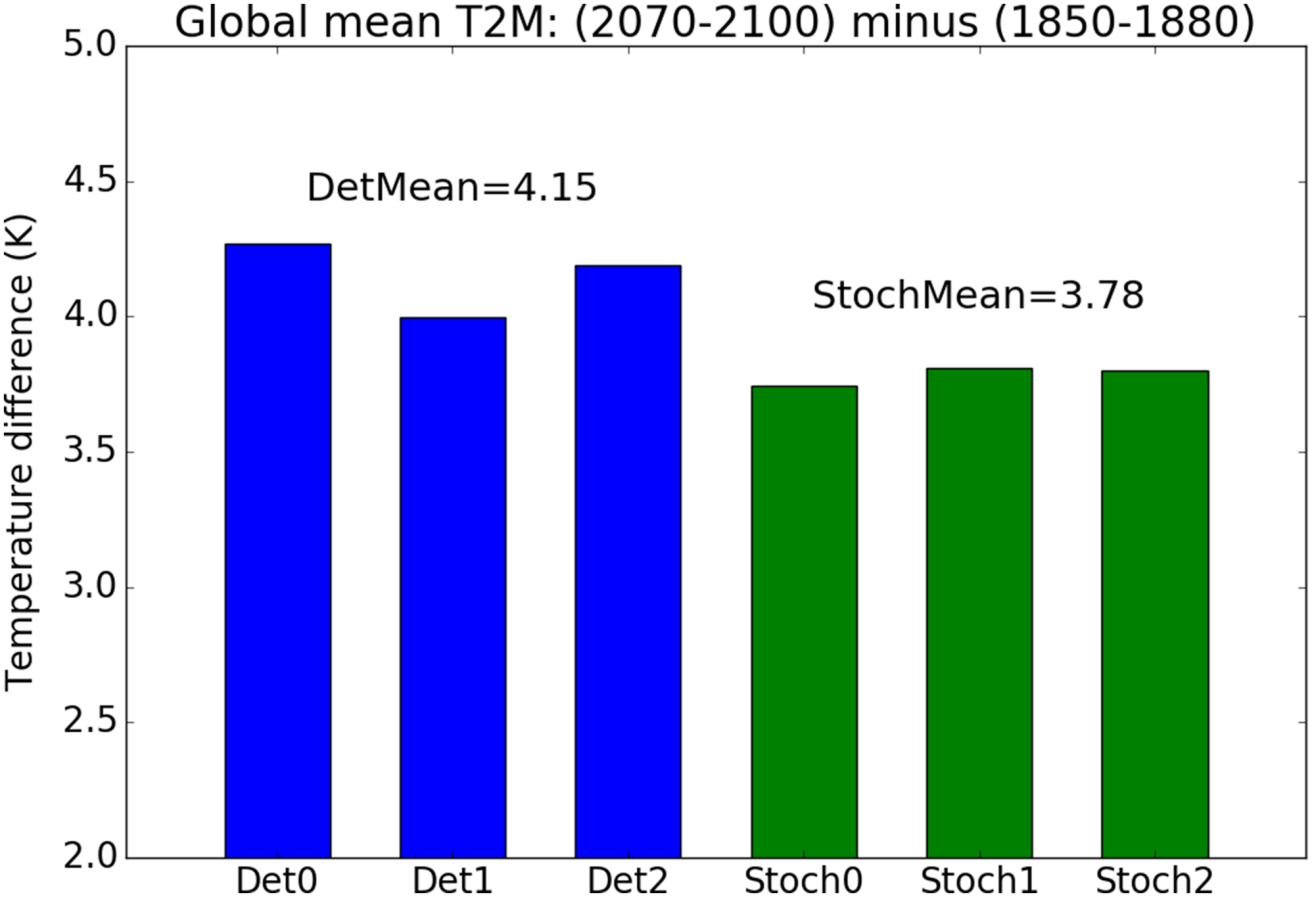
Extent of global waming by 2100 for three deterministic simulations (Det0, Det1, and Det2 in blue) and three stochastic simulations (Stoch0, Stoch1, and Stoch2 in green): global mean surface temperature over last 30 years of simulation (2070–2100) minus same over first 30 years (1850–1880). DetMean (respectively StochMean) denotes the mean of the three deterministic (respectively stochastic) values.

For a given SPHINX simulation, we measure the extent of (transient) global warming by taking the difference between the global mean surface temperature averaged over the first 30 years of the simulation (1850–1880) and the last 30 years (2070–2100). We will refer to this as the model's greenhouse gas sensitivity, or GHG sensitivity for short: It should not be confused with either the equilibrium climate sensitivity or transient climate response. Figure 1 shows the GHG sensitivity for the three deterministic and three stochastic simulations. It can be seen that all three deterministic simulations have a higher GHG sensitivity, at 4.15 K on average, than all three stochastic simulations, at 3.78 K on average. Therefore the stochastic scheme has reduced the projected global warming by about 10%.

**Figure 2:**
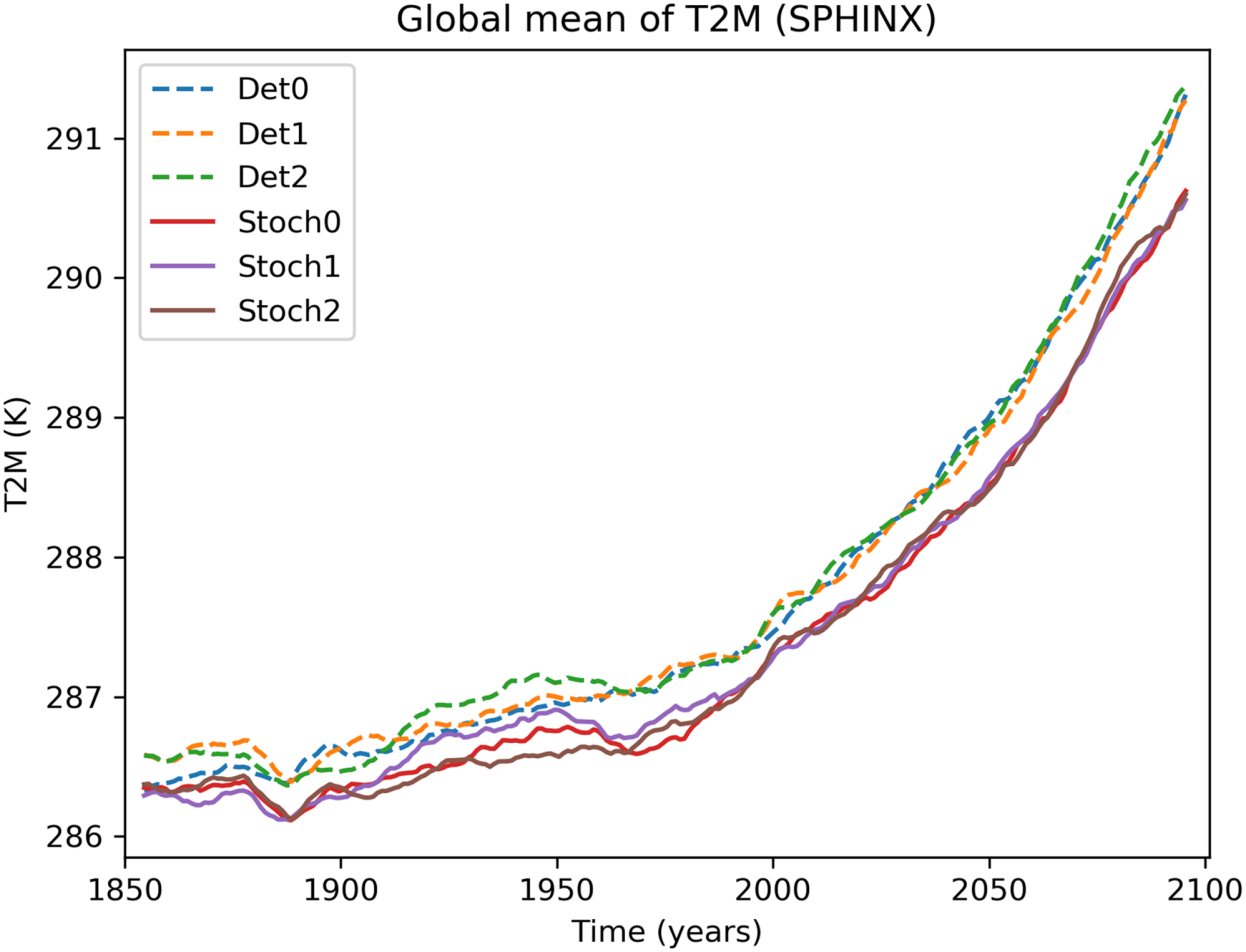
Evolution of global means of surface temperature (T2M) of the three deterministic simulations (Det0, Det1, and Det2: solid lines) and the three stochastic simulations (Stoch0, Stoch1, and Stoch2: stipled lines). A 10‐year running mean has been applied to smooth the data.

**Figure 3:**
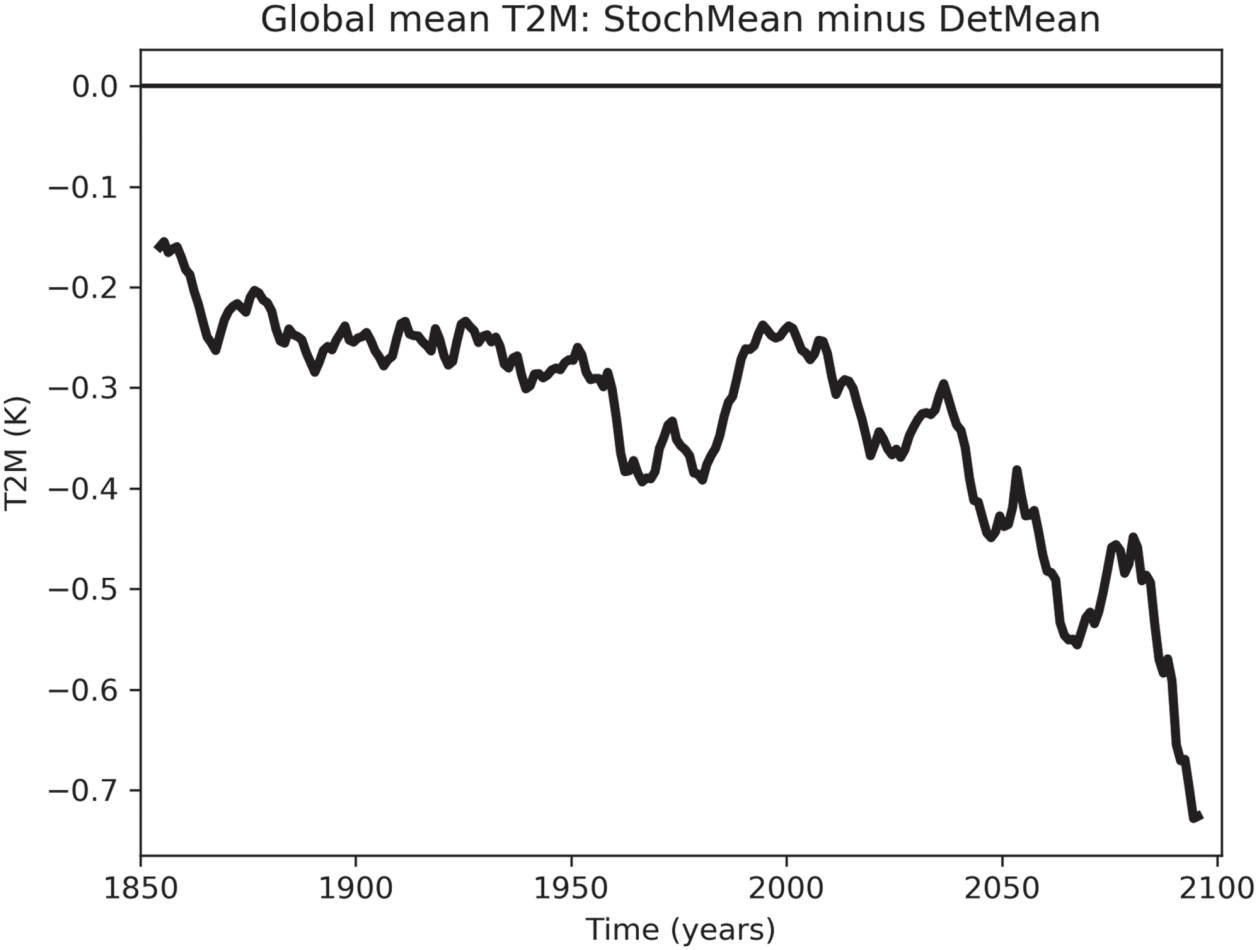
Time series of the difference in 10‐year running means of ensemble‐mean global mean temperature between the stochastic and deterministic simulations.

Figure 2 shows the temporal evolution of global mean surface temperature for all the individual simulations. A 10‐year running mean has been applied to isolate changes on climate timescales. The deterministic and stochastic ensembles are robustly separated across the entire time period, with the stochastic simulations having an overall cooler mean climate. Note that the separation is present already at the very start of the simulations: This is due to the fact that the two sets of simulations were initialized from their own individual “spin‐ups” and therefore are starting from different climates. The increased divergence that takes place as one approaches the end of the 21st century is more apparent in Figure 3, which shows the difference in the stochastic and deterministic ensemble means. During the twentieth century there is a fairly constant difference in mean surface temperature of around 0.3 K, which begins to grow rapidly from around 2040 onward. While emissions plateau toward the end of the 21st century in RCP8.5, there is no obvious sign that the divergence between the models has done the same. This may be simply due to the inherent lag in the system's response to forcing, but it also raises the possibility that the ultimate difference in equilibrium climate sensitivity may be greater than the 10% computed here.

**Figure 4:**
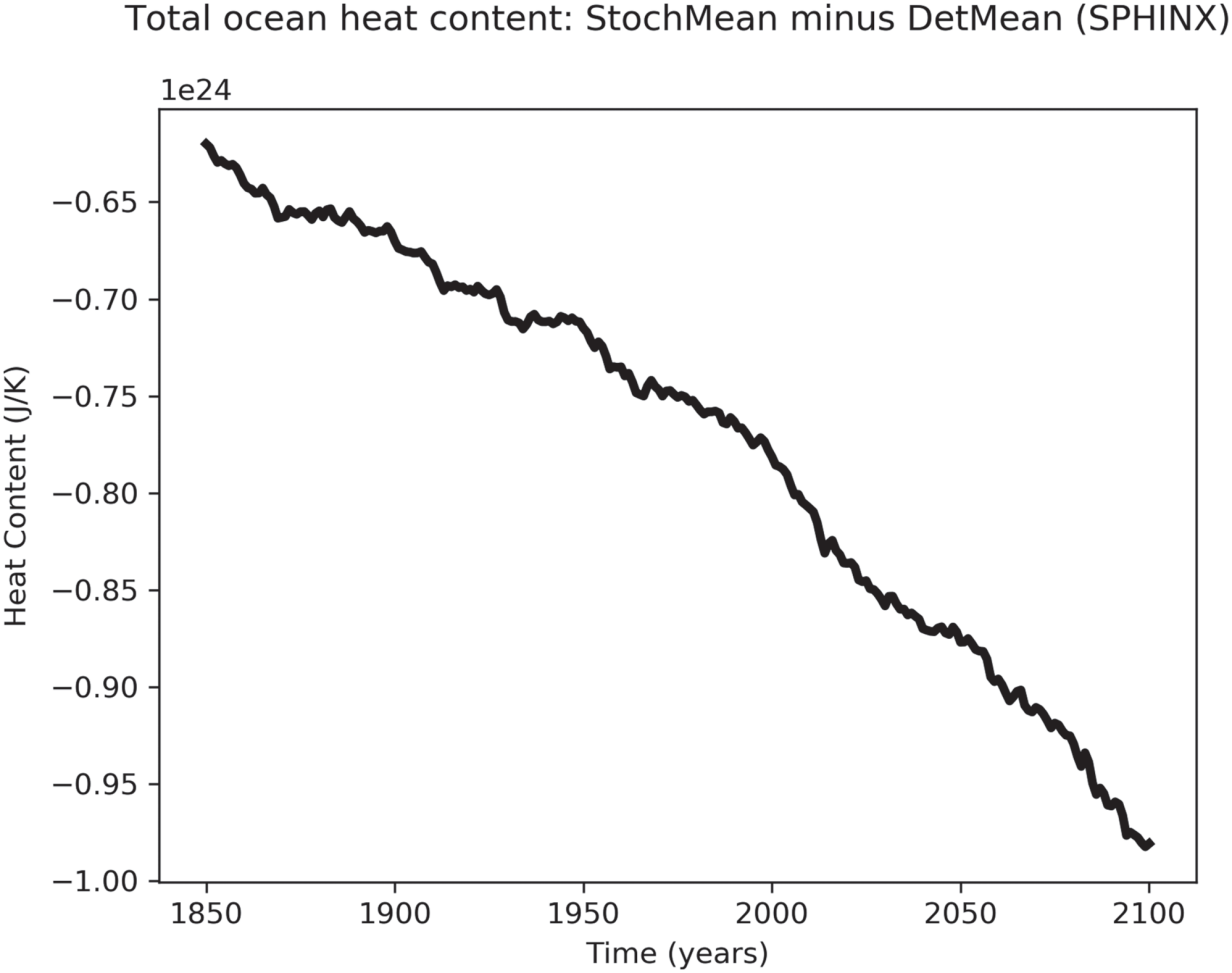
Time series of the difference in 10‐year running means of ensemble‐mean ocean heat content between the stochastic and deterministic simulations.

Figure 4 shows the difference between the stochastic and deterministic ensemble‐mean ocean heat content. In both simulations, the ocean heat content is increasing in the same manner as surface temperature (not shown). The fact that the difference is becoming more negative implies that SPPT is inhibiting the warming not just of the atmosphere but of the system as a whole. This also verifies that the results are not due to issues with the ocean spin‐up.

**Figure 5:**
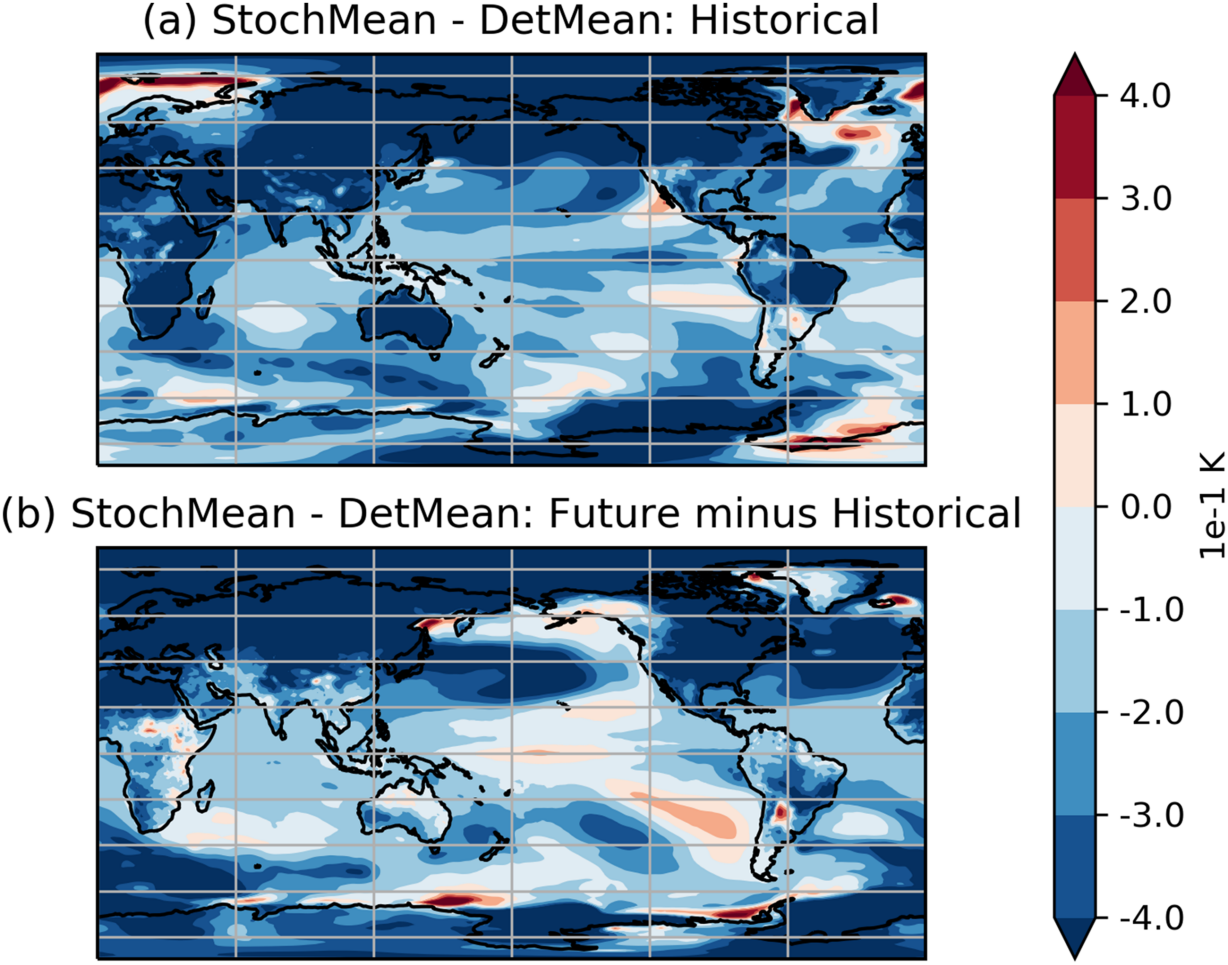
Evolution of mean surface temperature differences (K) between historical period and future period for the SPHINX experiments. In (a) stochastic ensemble mean (StochMean) minus deterministic ensemble mean (DetMean) over the period 1980–2010. In (b), the stochastic ensemble's mean warming between 1980–2010 and 2070–2100 minus that in the deterministic ensemble

Figure 5 shows the spatial pattern of the temperature differences in the historical period (1970–2000) and how this difference has changed at the end (2070–2100) of the simulation. It can be seen that the bulk of cooling with stochastic physics accumulates in the Northern Hemisphere, particularly over land and the Arctic. This tendency is amplified further by the end of the 21st century, with increased cooling relative to the deterministic model in the same regions. A possible explanation for this Northern Hemisphere accumulation is the phenomenon of Arctic amplification of global mean surface temperature changes due to anthropogenic forcing (Barnes & Polvani, 2015; Pithan & Mauritsen, 2014).

Through the rest of the paper we will aim to understand the drivers behind the evolution seen in Figure 3, including both the initial divergence leading to the cooler model climate with SPPT in the historical period, as well as the later increased divergence toward the end of the 21st century.

## 4 Analysis: Change in the Mean State

In this section we will aim to understand why the stochastic model has a cooler climate during the historical period.

Changes in global mean surface temperature (T2M) are associated with imbalances in the energy budget, since surface temperatures will change in order to bring the system to equilibrium. Therefore, we will take as our starting point the model's surface energy budget and use this to determine the dominant drivers of change when turning on SPPT.

**Figure 6:**
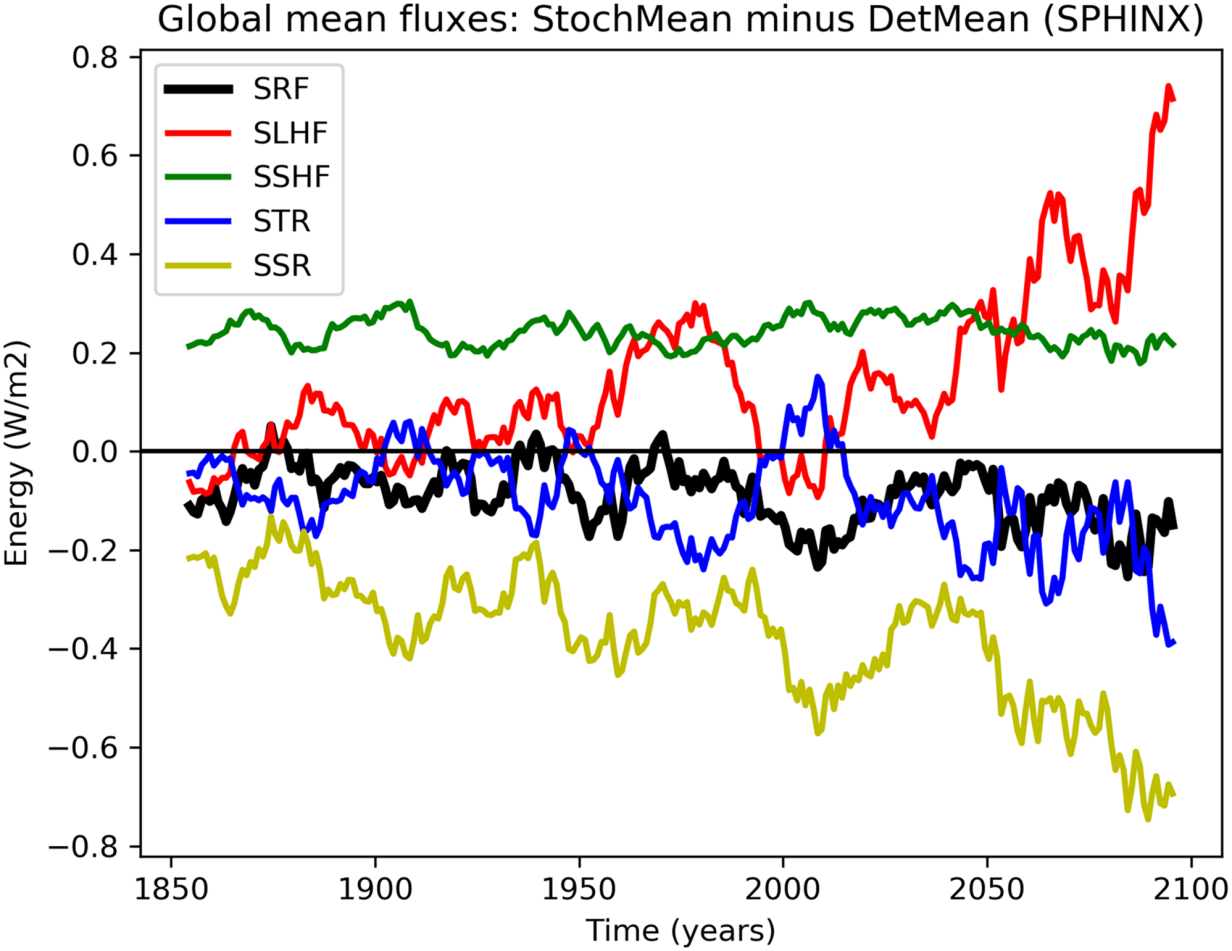
Evolution of surface energy fluxes (W/m^2^): stochastic ensemble mean (StochMean) minus deterministic ensemble mean (DetMean). Surface thermal radiation (STR: blue), surface solar radiation (SSR: yellow), downward surface latent heat flux (SLHF: red), downward surface sensible heat flux (SSHF: green), and net downward surface energy (SRF: black). Note we have used the EC‐Earth convention that downward fluxes are positive. Thus, SRF is the sum of other fluxes shown.

Figure 6 shows the differences in the fluxes making up this energy budget between the stochastic and deterministic ensemble means. Note that we have used the convention that downward fluxes are positive. In particular, an increase in downward latent heat flux due to SPPT corresponds to a *decrease* in evaporation (an upward flux), and vice versa. The upshot of this convention is that the net surface energy can be conveniently obtained by adding up the other fluxes, and so the dominant source of change can be identified at any point in time. As before, we have smoothed the time series by a 10‐year running mean, in order to retain only the fluctuations taking place on climate timescales.

During the twentieth century the mean difference in the net flux is around −0.1 W/m^2^, which grows to around twice that by the end of the 21st century, consistent with both the cooler mean climate and the reduced GHG sensitivity. Anthropogenic forcings lead to a net positive surface energy imbalance for both configurations, which both sets the historical mean temperature, and, as this imbalance increases throughout the RCP8.5 scenario, the extent of global warming. The reduction of this excess amount of energy due to SPPT in the historical period leads to a cooler historical climate, and the growth of this reduction implies a weaker GHG sensitivity.

We will begin by trying to understand the initial lower average net surface energy flux and then follow up by trying to understand the decreased GHG sensitivity.

### 4.1 Historical Energy Budget Analysis

Figure 6 shows that the dominant contribution to the decreased surface energy flux due to SPPT is a strong decrease in surface solar radiation (SSR). The second consistent, but lesser, contribution is made by a decrease in surface thermal radiation (STR), associated with a decrease in the amount of longwave radiation emitted to the surface by the atmosphere. This is likely due to a decrease in the global mean atmospheric water vapor content due to SPPT (not shown), which reduces the greenhouse effect. However, as will be seen in the next section, this change appears to be a *response* to the cooling induced by reduced solar radiation, so we do not discuss the role of water vapor further.

Changes in net solar radiation will be determined by changes to the albedo of the surface, the cloud coverage, and the albedo of the clouds. This latter property, a function of the optical thickness of the cloud, depends to a large extent on the CLWC of the clouds, that is, the total amount of liquid water per unit volume of air in a cloud parcel; this is sometimes also referred to as the “liquid water path” (Han et al., 1998). Changes in surface albedo are on the other hand going to be associated with changes in snow and ice cover; EC‐Earth does not have dynamic vegetation, so the albedo of the land will not otherwise change. By decomposing the net SSR into a contribution from the central region 60°S to 60°N and a high‐latitude region (the combined regions 60–90°N and 60–90°S), one finds that ∼86% of the difference in SSR due to SPPT can be accounted for by differences in 60°S to 60°N, where surface albedo changes are small. This suggests that changes in sea ice and snow coverage are not primarily responsible. Computing time series of global mean sea ice volume and snow coverage supports this conclusion, showing no notable differences between the stochastic and deterministic simulations during the entire historical period (not shown).

**Figure 7:**
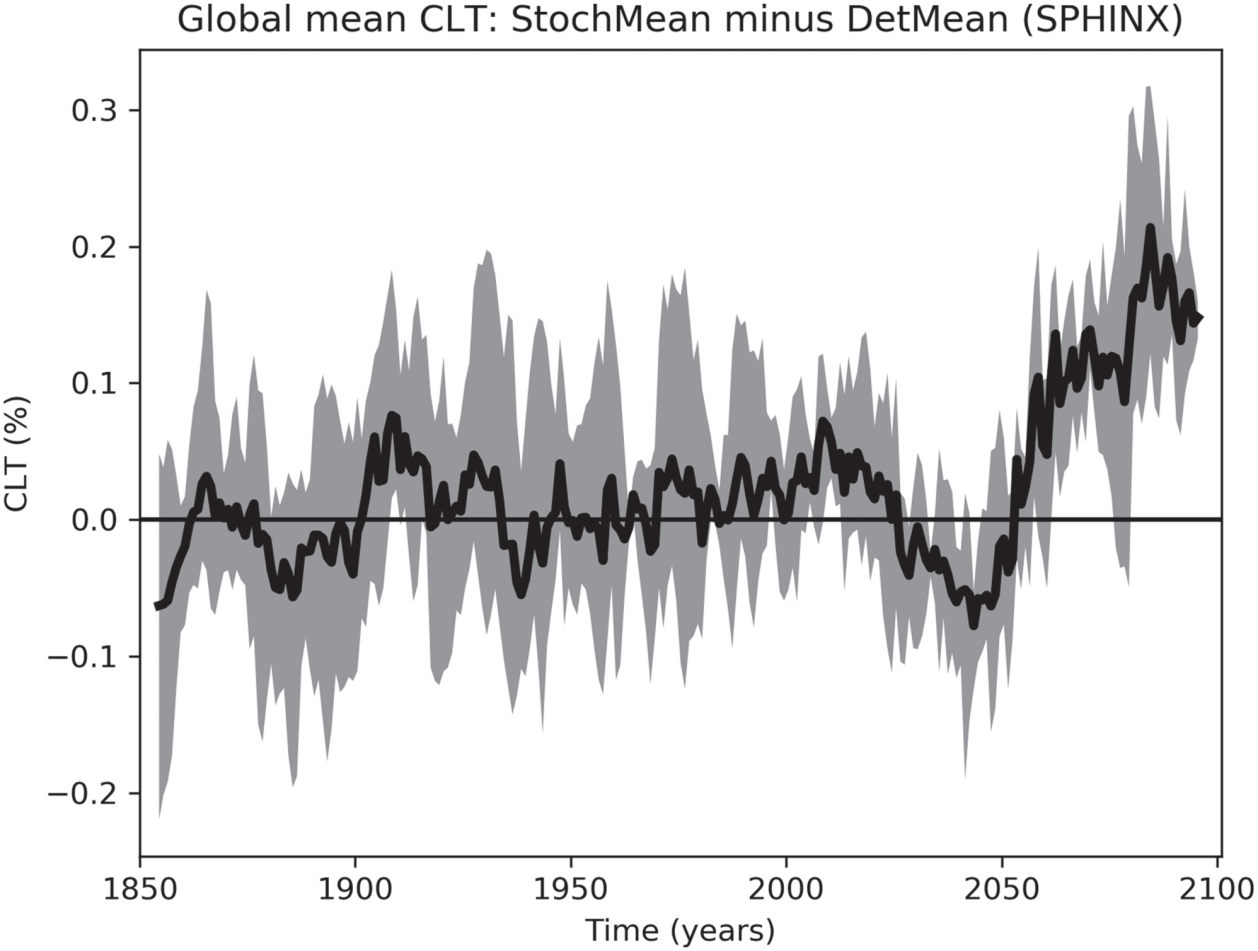
Evolution of total cloud cover (TCC) for the SPHINX experiments: stochastic ensemble mean (StochMean) minus deterministic ensemble mean (DetMean). A 10‐year running mean has been applied to smooth the time series. Shading indicates the maximum/minimum difference attained across all three pairs.

**Figure 8:**
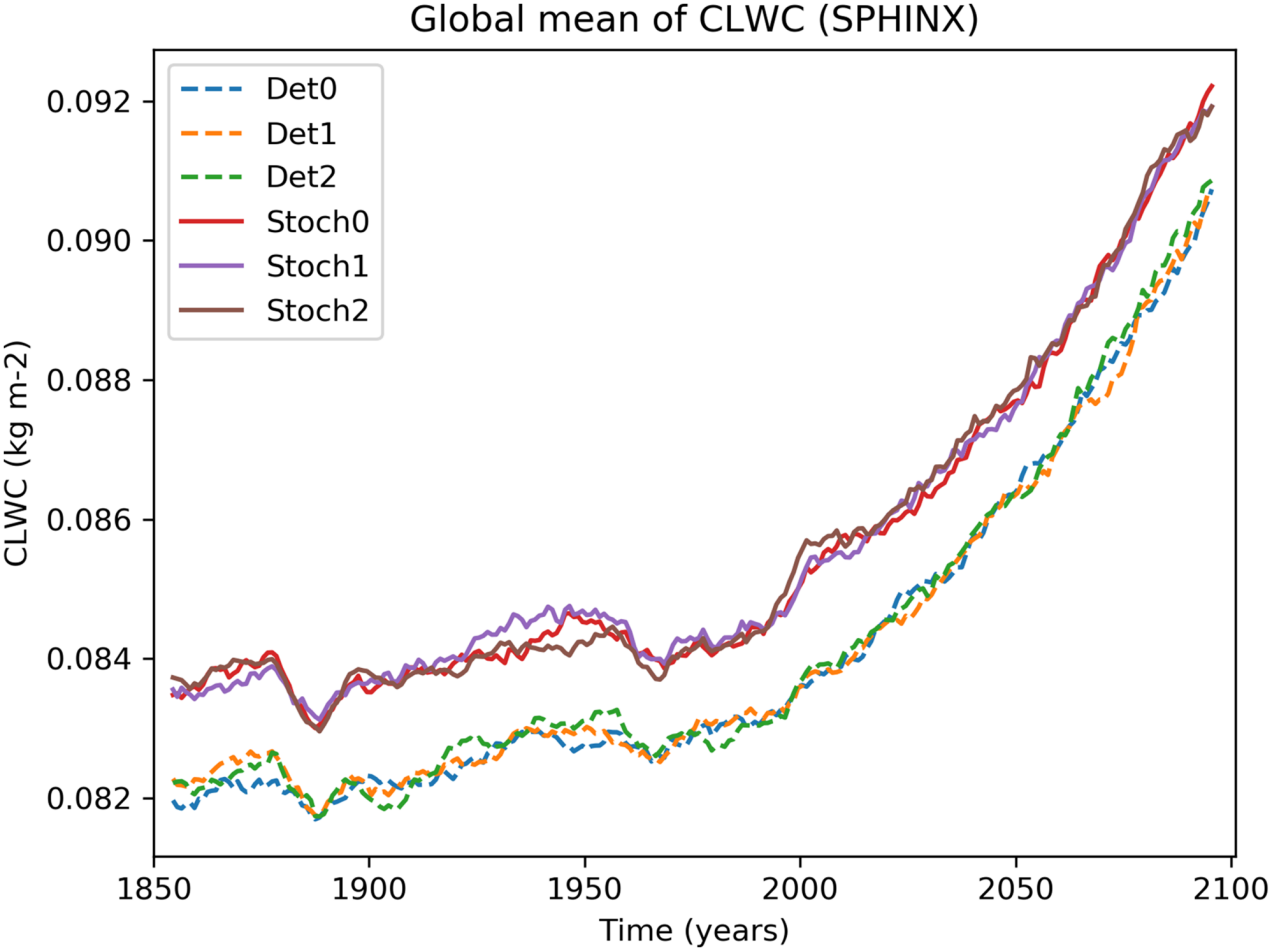
Evolution of vertically integrated cloud liquid water content (CLWC) for the SPHINX simulations. A 10‐year running mean has been applied to smooth the time series.

**Figure 9:**
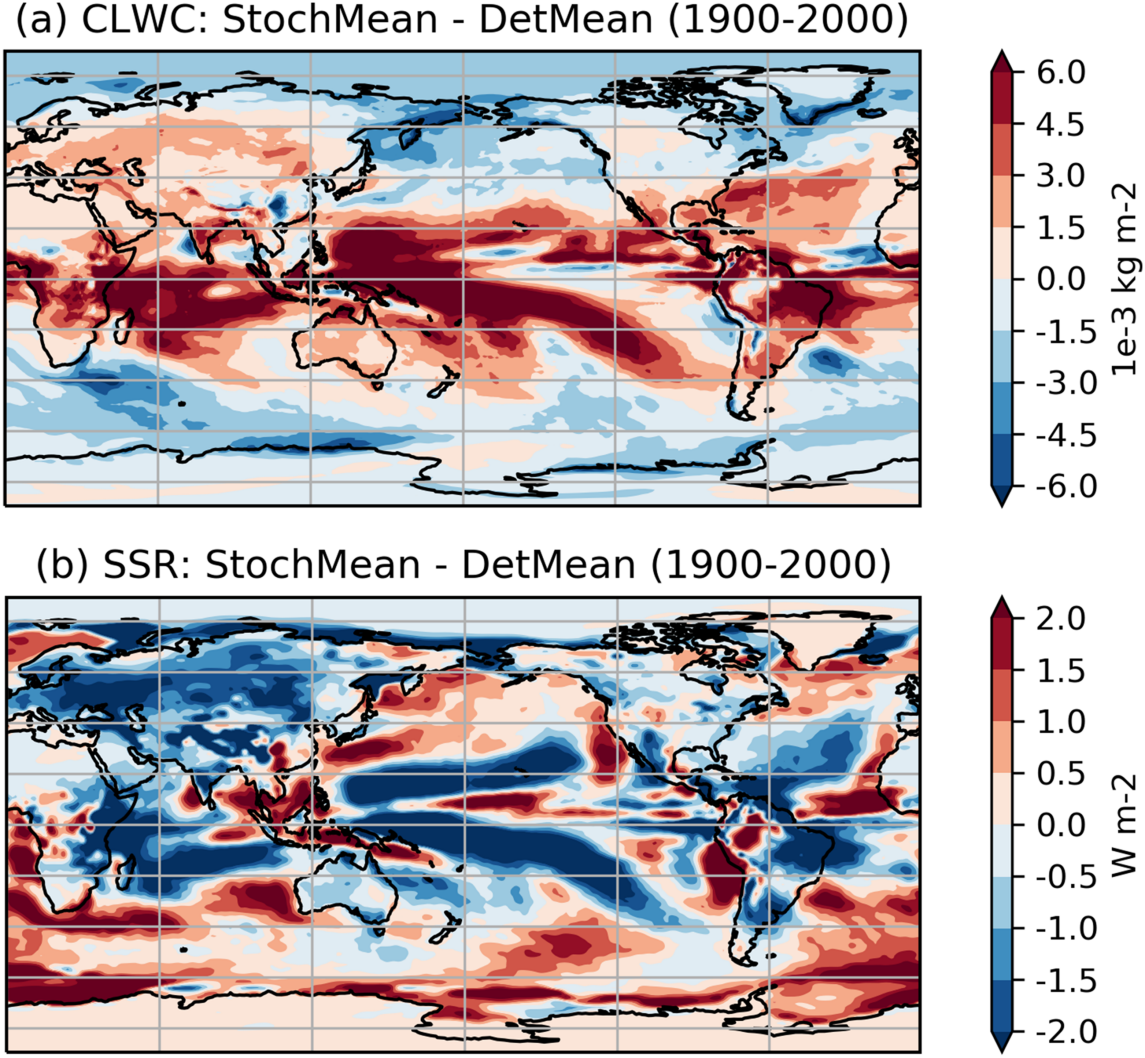
Stochastic minus deterministic ensemble mean (1900–2000) for (a) cloud liquid water content (CLWC) and (b) surface solar radiation (SSR). SPHINX experiments.

Turning then to changes in cloud coverage, Figure 7 shows that between 1850 and 2000 there is no notable change in the total cloud cover (TCC) between the two ensembles. On the other hand, Figure 8 shows a robust increase in CLWC with SPPT across the entire simulation period. The difference is approximately constant in time and represents an increase of about 1.6% relative to the deterministic mean; this is consistent with the findings of Strømmen et al. (2019), who find a similar increase in CLWC in a different version of EC‐Earth. Figures 9a and 9b show the spatial structure of CLWC and SSR changes, respectively. The majority of the CLWC change is concentrated in the tropical Pacific and the Indian Ocean. In these regions there is good spatial coherence between CLWC and solar radiation changes, with a pattern correlation between the two fields, when restricted to 60°S to 60°N, of around −0.63. It is known that the dependence of cloud albedo on CLWC is nonlinear, with the impact being stronger for optically thin clouds than for optically thick clouds

(e.g., Han et al., 1998), so correlations close to −1 would not necessarily be expected. Furthermore, while the global mean cloud cover does not change with SPPT, there are various regional changes (not shown) which may further influence the exact location of solar radiation changes. We conclude that it is likely that the increased CLWC is a major cause of the decreased solar radiation.

To assess the model's global mean CLWC, we compared against observational data obtained via remote sensing techniques (Wentz et al., 2012). The data covered the period 2000–2008 and were restricted to ocean points only; by restricting the model data similarly, we found that the deterministic model has a positive bias (too much cloud water) of around 10%. While the stochastic models have increased this bias, the effect is small relative to this initial bias. It is also important to note that the deterministic model was tuned to achieve a realistic energy budget for the historical period, which may have helped to adjust the CLWC toward a more realistic value. So it may be the case that the stochastic model would have a similar or smaller bias if it were also tuned.

The increased CLWC is present already at the start of the SPHINX simulations, implying that this change was stably in place after the model's spin‐up phase. Therefore, we will turn to the FastSPHINX experiments to assess this change further. In particular, we want to determine if CLWC changes are a response to some other rapid mean‐state change.

### 4.2 Determination of Fast Changes Due to SPPT

**Figure 10:**
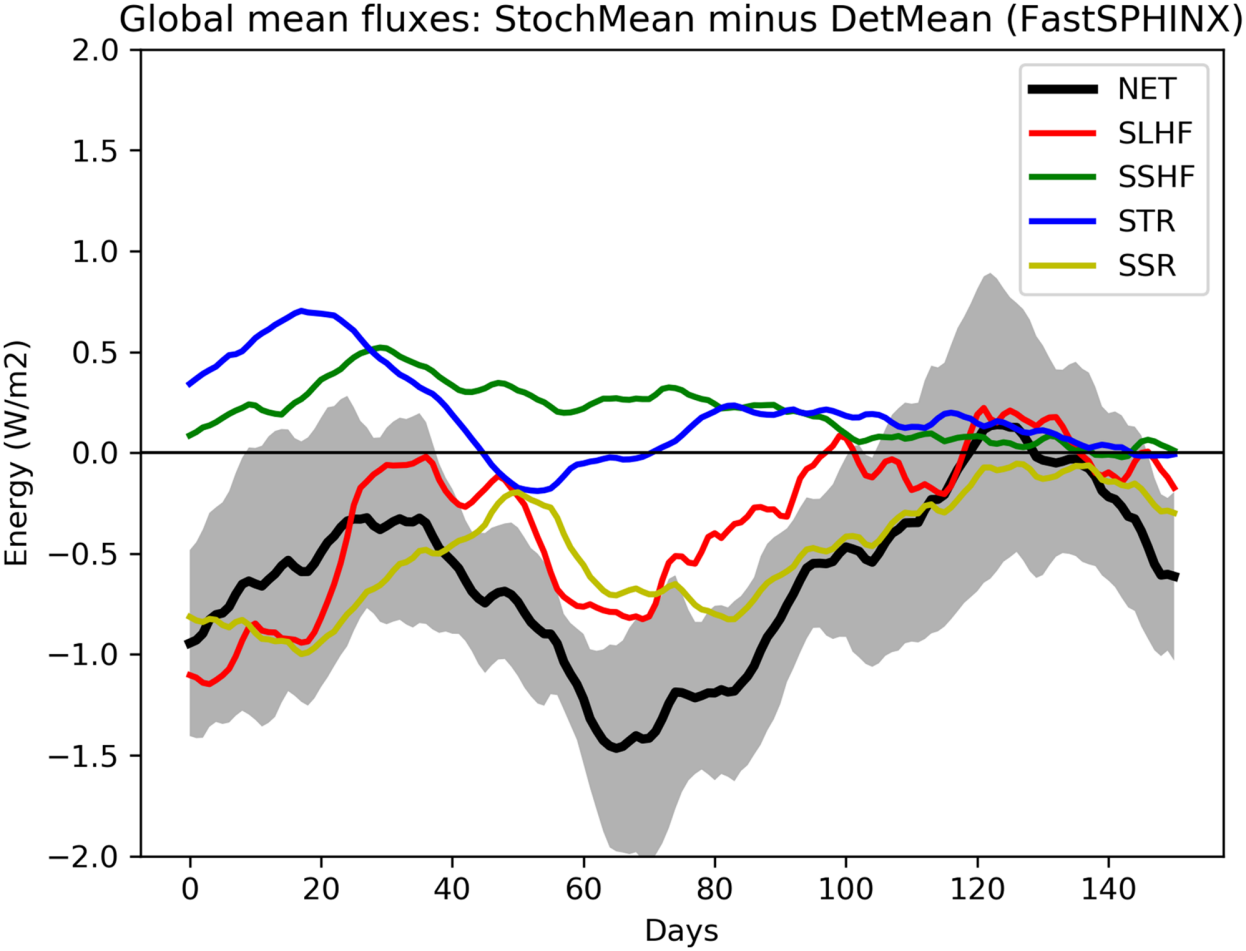
Evolution of global mean surface energy fluxes for the FastSPHINX experiments: stochastic ensemble mean (StochMean) minus deterministic ensemble mean (DetMean). Surface latent heat flux (SLHF: red), sensible heat flux (SSHF: green), thermal radiation (STR: blue), solar radiation (SSR: yellow), and net surface energy (SRF: black). The shading indicates the standard error of the mean estimate at every timepoint. A 30‐day running mean has been applied to smooth all time series.

As explained in section 2.4, the FastSPHINX experiments will allow us to identify the rapid response to turning on SPPT. Figure 10 shows the rapid change in surface energy fluxes: A 30‐day running mean has been applied to reduce noise and highlight the more systematic trends. It can be seen that SPPT has decreased SRF, with the mean difference over the 6‐month period being −0.6 W/m^2^ . This is larger than the 0.1–0.2 W/m^2^ seen in the long climate experiments, but this is likely just reflecting the fact that it takes the model many years to reach its new equilibrium when SPPT is turned on. The dominant contribution to this decrease is a reduction in both net solar radiation and latent heat flux, with the latter (due to the sign convention) corresponding to an *increase* in evaporation. Surface temperatures rapidly cool as a result of this drop in surface energy, before temporarily warming again as the system begins to respond (not shown). As we know from the SPHINX simulations, eventually a new, cooler equilibrium is reached.

**Figure 11:**
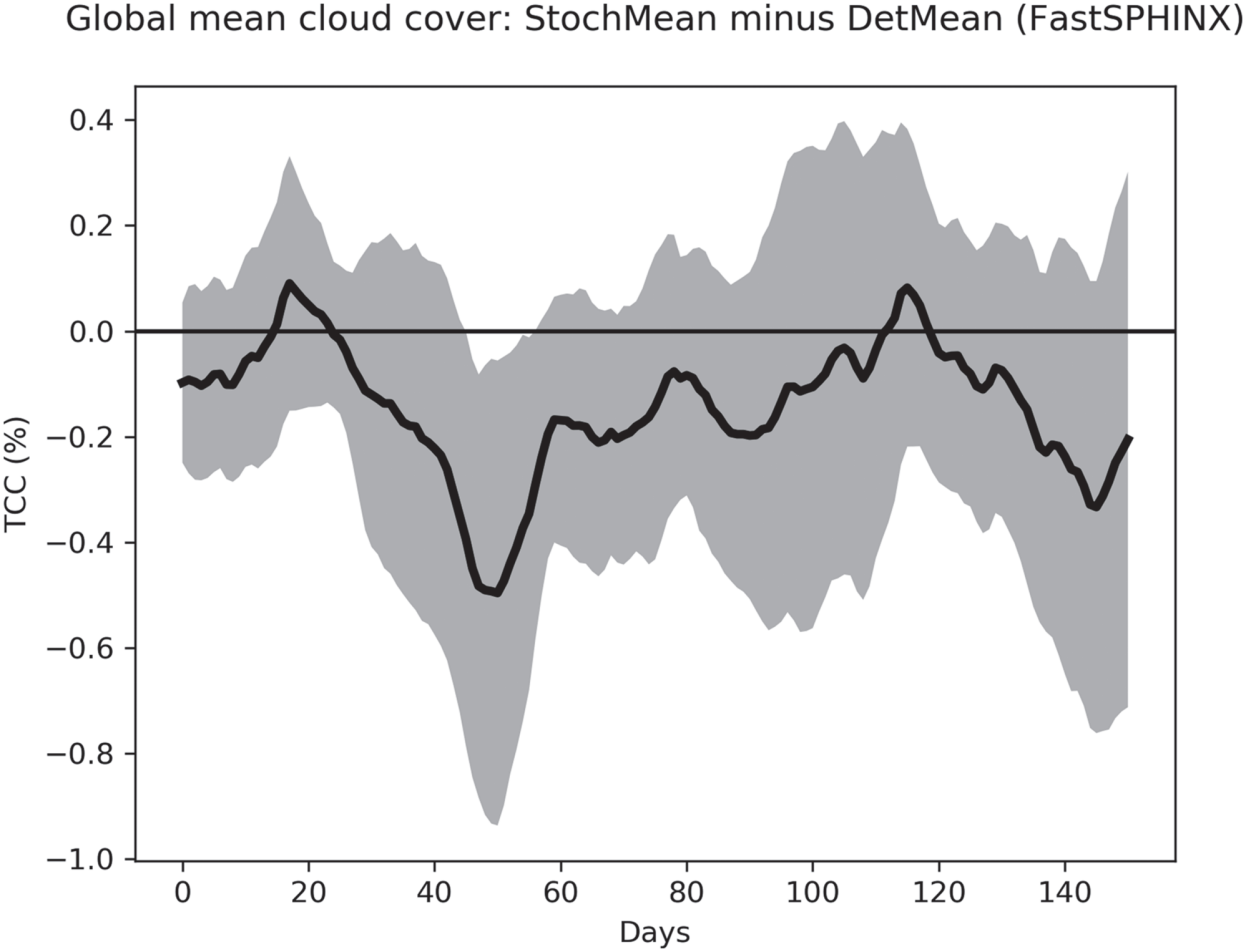
Evolution of total cloud cover (TCC) for the FastSPHINX experiments: stochastic ensemble mean (StochMean) minus deterministic ensemble mean (DetMean). A 30‐day running mean has been applied to smooth the time series. The shading indicates the standard error of the mean estimate at every timepoint.

**Figure 12:**
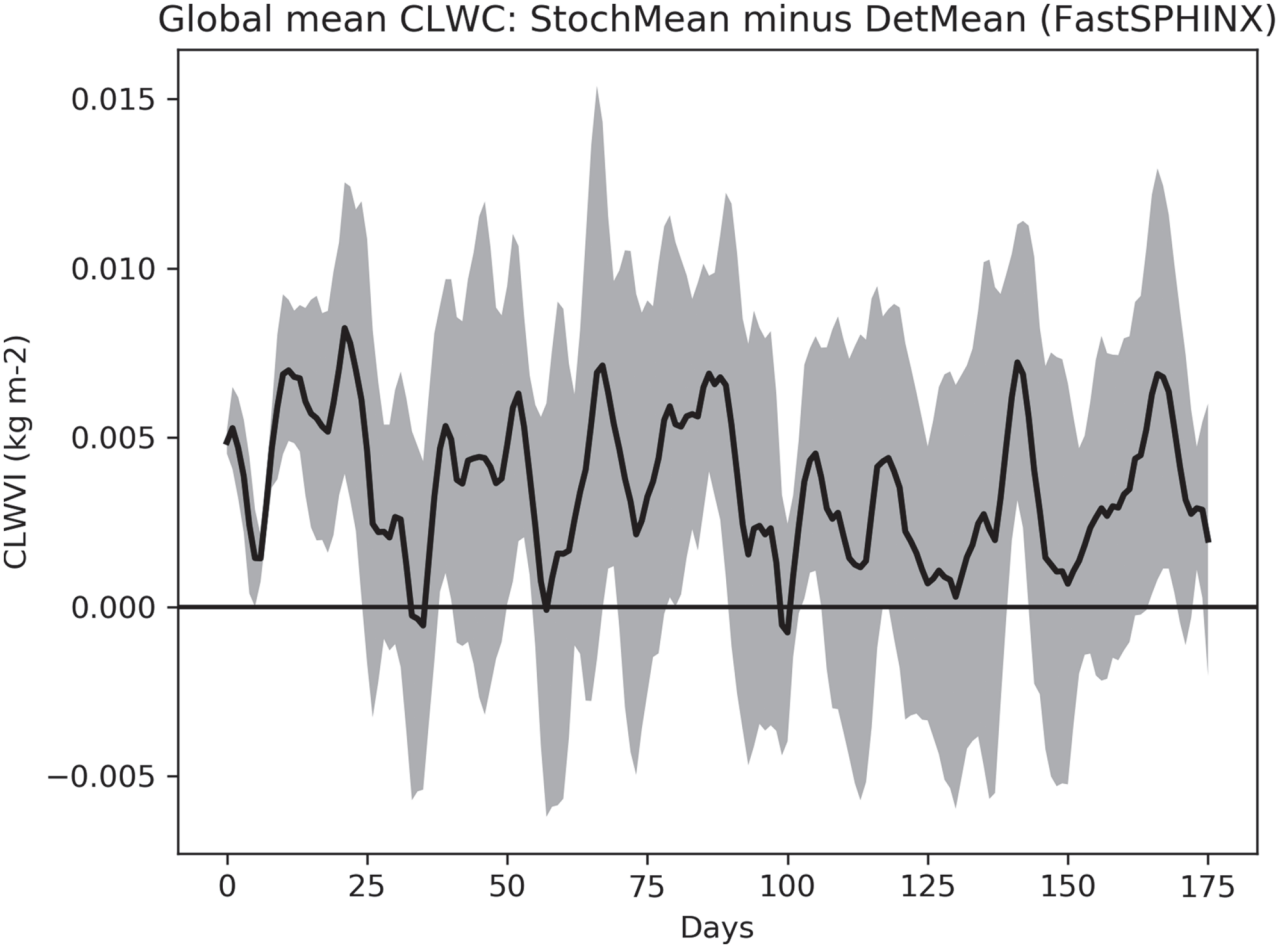
Evolution of vertically integrated cloud liquid water content (CLWC) for the FastSPHINX experiments: stochastic ensemble mean (StochMean) minus deterministic ensemble mean (DetMean). A 5‐day running mean has been applied to smooth the time series. The shading indicates the standard error of the mean estimate at every timepoint

**Figure 13:**
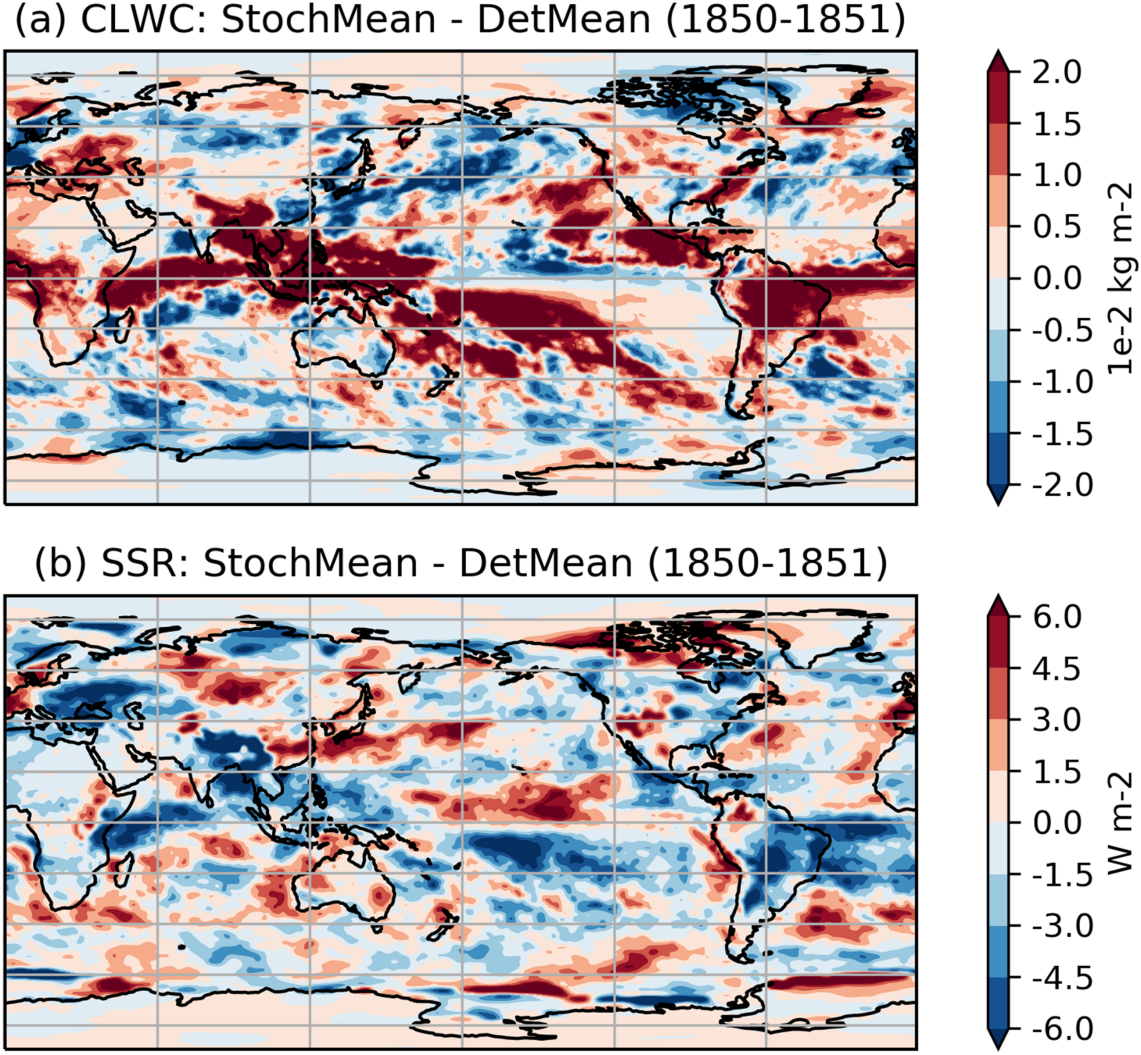
Stochastic minus deterministic ensemble mean (1850–1851) for (a) cloud liquid water content (CLWC) and (b) surface solar radiation (SSR), in the FastSPHINX experiments.

**Figure 14:**
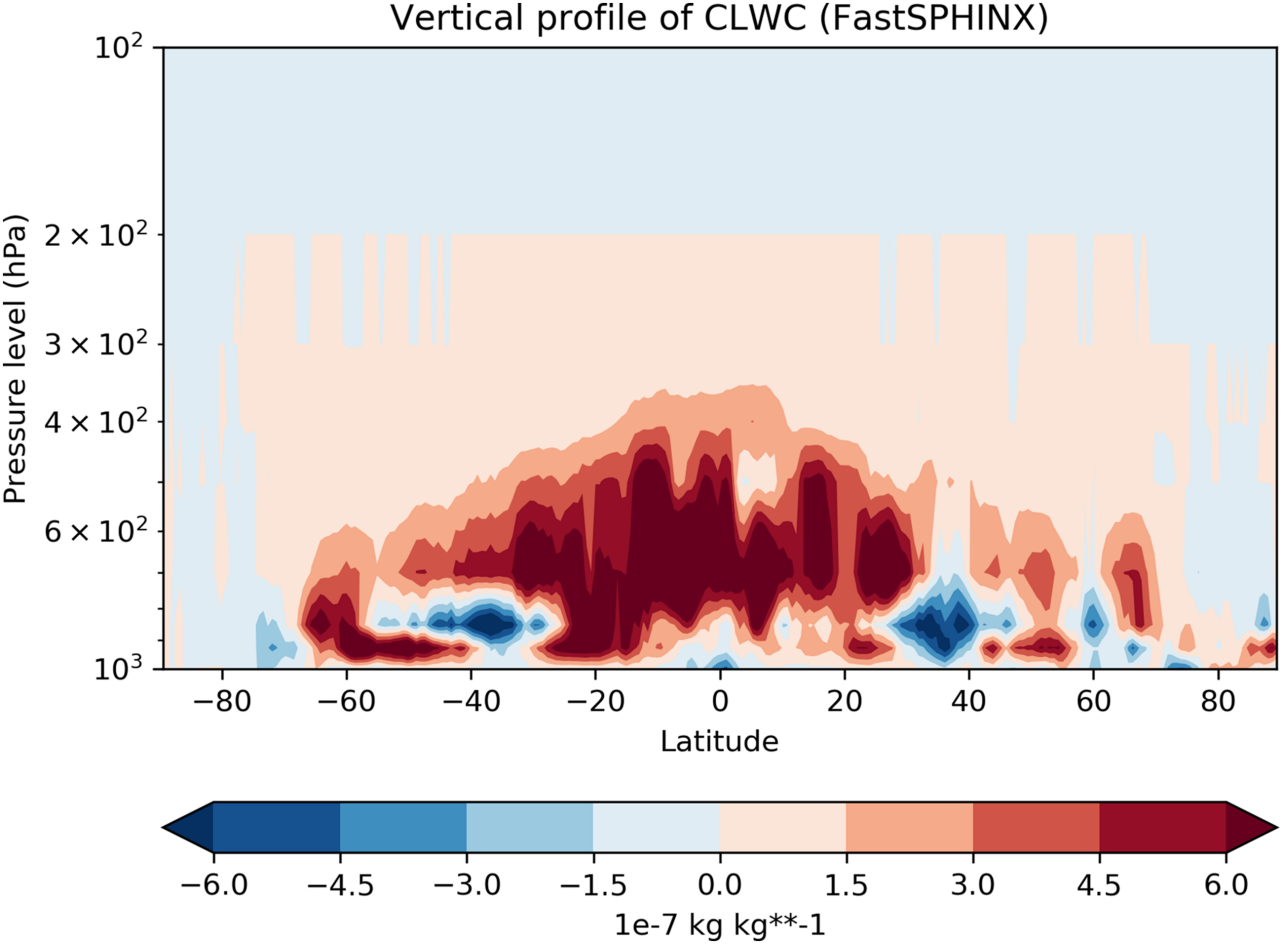
Cloud liquid water content (CLWC) as a function of latitude and pressure for the FastSPHINX experiments: stochastic ensemble mean (StochMean) minus deterministic ensemble mean (DetMean). Note the logarithmic scale of the *y* axis.

Let us first discuss solar radiation changes. Figure 11 shows the rapid changes in TCC, showing a slight trend for the stochastic model to have reduced cloud cover compared to the deterministic model. Because such a decrease would be expected to lead to an increase in net solar radiation, we conclude that, as with the SPHINX simulations, changes in cloud cover cannot explain the reduced solar radiation. Figure 12 shows the evolution of CLWC differences. A coarser, 5‐day running mean has been applied to highlight the speed of the change: The increase with SPPT, of around 2.5%, is robust and in place within days. In fact, we find that the deterministic and stochastic simulations are statistically significantly separated in terms of their global mean CLWC within the first 6 hr (not shown). The spatial pattern of the changes is similar to those seen in the SPHINX data, as seen in Figure 13, which shows spatial changes averaged over the entire 6‐month period for CLWC and SSR. As in SPHINX, the SSR changes correlate well with the CLWC changes, with a pattern correlation of −0.67 between 60°S and 60°N. Cloud water on individual pressure levels was not available for the SPHINX experiments but was output from the FastSPHINX simulations. Figure 14 shows the vertical structure of the changes, as a function of latitude and pressure. The increase is greatest between 400 and 800 hPa and is less robust near the surface. This may be related to the fact that the stochastic perturbations are tapered to zero as one approaches the surface. From about 800 hPa and upward the increase is firmly in place and, while greatest in the tropics, extends across the full latitudinal range.

Next, we consider the other major contributor to the reduced surface flux, namely, the increased evaporation with SPPT. In the IFS, the amount of evaporation at a grid point depends primarily on the surface wind speeds and the extent to which the specific humidity at the surface grid point differs from the saturation humidity (a function of surface temperature). There is also a contribution from turbulent processes, which depend on surface roughness (see ECMWF, 2017), but because surface roughness does not differ between the deterministic and stochastic simulations, this effect can be safely ignored. We may therefore assess the evaporation changes expected due to changes in wind speeds with fixed humidity gradient and those expected due to changes in humidity gradient with fixed wind speeds. One finds in this way that the dominant source of the increased evaporation with SPPT is due to a small but consistent increase in surface wind speeds (not shown). This increase in wind speeds is also a rapid response, coming into effect within ∼1 day. However, evaporation changes do not lead changes in cloud water and, after a sufficiently long spin‐up, become negligible (see the data Figure 6 before ca. 1950). This suggests that the impact of increased wind speeds are eventually canceled out by the surface cooling, which reduces the saturation humidity and, therefore, evaporation.

Finally, note that the trend in STR is, if anything, toward a slight increase with SPPT. This implies that the reduction in this quantity seen in Figure 6 is a long‐term response to other forcings, rather than a driver of such changes. Only solar radiation changes persist from the very start of the FastSPHINX experiments through the spin‐up and the subsequent SPHINX experiments.

These results were found to be robust across both sensitivity experiments performed (described in section 2.4). In the forced SST simulations, the changes in the energy budget were consistent with those seen in the coupled experiments. In particular, both sets of simulations showed the same increase in cloud liquid water and resulting decrease in SSR. In the simulation with SPPT turned on, but the “humidity fix” turned off, the rapid response of the model is the same as that seen with the “fix” turned on, namely, a sharp increase in cloud liquid water. However, the eventual drying of the atmosphere, which takes place without the “fix,” is, by magnitude, a much bigger change than this increased cloud water. The reduced availability of vapor for condensation thus eventually dominates, ultimately reducing the global mean cloud water content. However, because the ratio of total column cloud water to total column water vapor remains approximately the same whether the “fix” is on or not, we conclude that the “fix” is not likely to be responsible for the differences found between the deterministic model and the version with SPPT including the “fix.”

In conclusion, we find that the dominant impact to the models energy budget when turning on SPPT is to decrease the net SSR by way of a rapid increase in cloud liquid water. This increases cloud albedo, thereby reducing incoming solar radiation and, consequently, cooling the surface. The CLWC increase may be due to the stochastic perturbations interacting with the highly nonlinear process of condensation. Given a parcel of air close to saturation, a perturbation of temperature or humidity in one direction may cause the parcel to condense its water, increasing the total CLWC. However, a perturbation in the opposite direction would in this situation result in no change in total CLWC. An important point here is that the implementation of SPPT used (Palmer et al., 2009) has a “supersaturation limiter” in place, which prevents this nonlinear effect from taking place within a single time step: If the stochastic perturbations leave a grid point in a supersaturated state, the limiter ensures that these perturbations are not applied. Nevertheless, if the SPPT scheme pushes a parcel close to saturation, the actual model dynamics may, on the subsequent time step, trigger condensation. In general, by broadening the distribution of humidity tendencies, one may expect to see more condensation triggered on average. Therefore, given a systematic application of symmetric, mean‐zero perturbations to the tendencies, as is done with SPPT, a change in the mean CLWC is plausible.

## 5 Analysis: Change in GHG Sensitivity

In the above analysis we linked the changes in the mean state to rapid changes in the model's cloud properties, in particular the mean CLWC. In this section we aim to explore possible mechanisms that might explain the reduction in GHG sensitivity.

Figure 6 shows that the decrease in solar radiation due to SPPT increases as the simulations progress and remains by far the dominant source of decreased net surface energy. Given that the dominant component of spread in climate sensitivity estimates across general circulation models (GCMs) is due to differences in cloud feedbacks (Dufresne & Bony, 2008), it is plausible that these may explain the reduced GHG sensitivity with SPPT. Cloud feedbacks can be decomposed into a contribution from three critical cloud properties: cloud altitude, cloud coverage, and cloud optical depth (Ceppi et al., 2017; Zelinka et al., 2012a). The primary impact on cloud altitude changes is on longwave radiation, while cloud cover and cloud optical depth changes are the primary sources of shortwave changes. Because changes in the shortwave radiation dominate the energy budget, we will focus on cloud cover and cloud optical depth changes: The latter is, as discussed previously, going to be largely due changes in CLWC.

We now discuss the two key cloud feedbacks in turn, as well as the possible impact of nonlinearity in climate sensitivity feedbacks.

### 5.1 Differences in Cloud Cover Feedback

**Figure 15:**
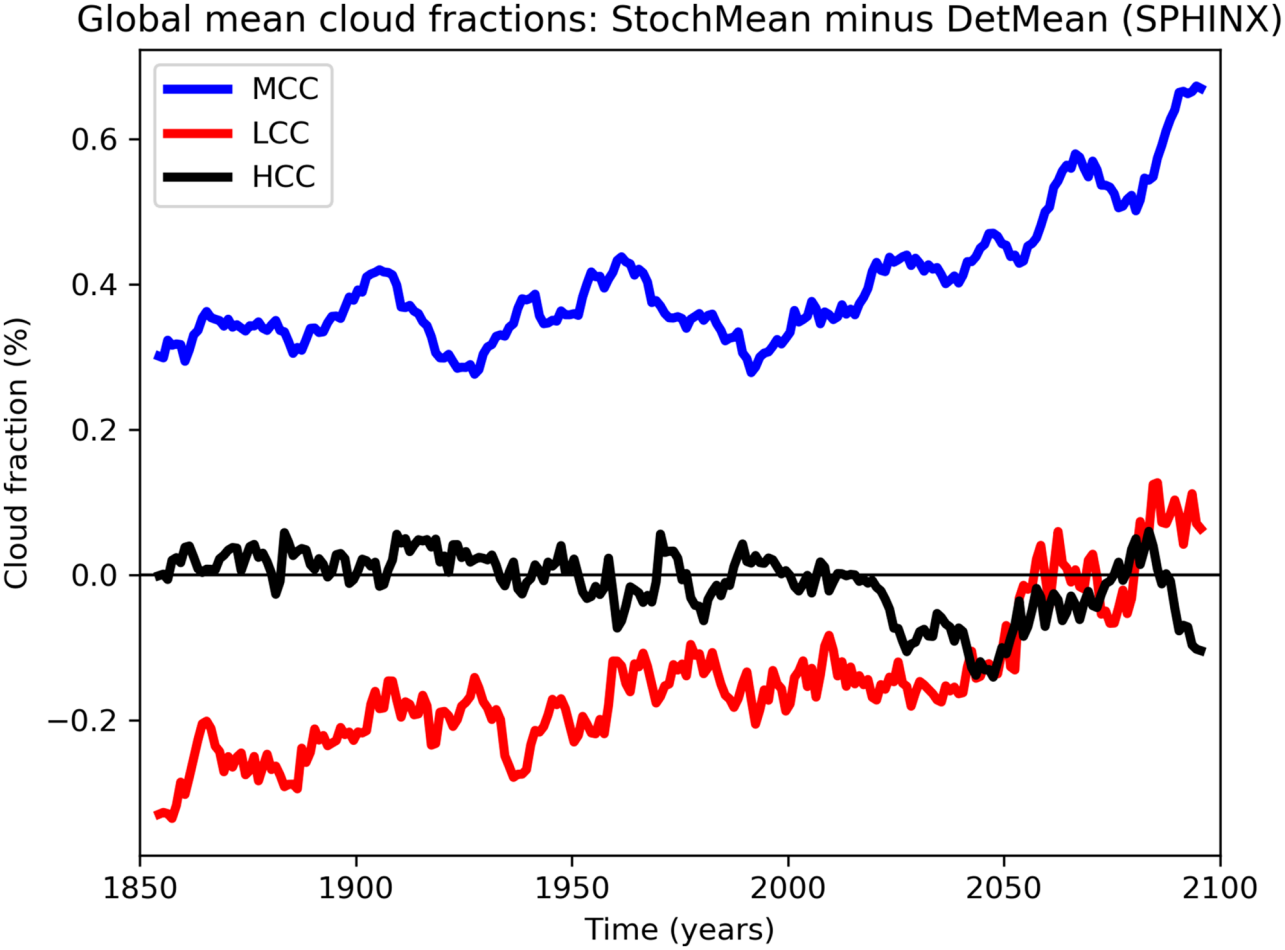
Evolution of cloud cover in the SPHINX simulations (1850–2100): stochastic ensemble mean (StochMean) minus deterministic ensemble mean (DetMean) for low‐level cloud cover (LCC), midlevel cloud cover (MCC), and high‐level cloud cover (HCC).

Figure 7 shows that while the stochastic and deterministic simulations do not differ notably in TCC up to around 2050, after this date there is a robust change. For all simulations, deterministic and stochastic alike, the TCC drastically reduces as global warming intensifies (not shown). Figure 7 shows that the difference between the stochastic and deterministic simulations is becoming more positive, implying that this reduction in cloud is taking place at a slower rate with SPPT turned on. Figure 15 shows again the difference in global mean cloud cover, but stratified now into low‐, medium‐, and high‐level clouds. SPPT has reduced low cloud cover and increased medium cloud cover, thereby effectively raising the average cloud altitude. We also see from this figure that the less rapid decline in TCC with SPPT is due to a less rapid decline in low‐ and middle‐level clouds. All three cloud types reduce as the model warms.

It is not straightforward to diagnose the cause of this reduced rate of low cloud breakup. While GCMs frequently show a decrease in low cloud cover with global warming (e.g., Zelinka et al., 2012b, 2012a), the causes for this are not well understood. Bretherton and Blossey (2014) discussed one potential explanation (applicable to cumulus‐under‐stratocumulus boundary layers and stratocumulus topped mixed layers), which they dubbed the “entrainment‐liquid‐flux adjustment” mechanism. In this framework, the immediate response of a cloud to an instantaneous warming is to increase the liquid water flux everywhere in the cloud. This triggers an increased buoyancy flux, hence increased cloud‐layer turbulence, which has the effect of thinning the cloud through the entrainment of warm, dry air through the cloud. With SPPT turned on, the clouds have a larger amount of liquid water in them to start with, and it is possible that this could reduce the relative impact of such an entrainment‐liquid‐flux response. Both theory and Large Eddy Simulation (LES) studies generally support the role of cloud water in maintaining stratocumulus clouds, and how the depletion of moisture plays a role in both the thinning of these clouds and their transition to scattered cumulus clouds with sparser coverage (Sandu & Stevens, 2011; Wood, 2012). Again, the greater availability of cloud water in the stochastic simulations may slow down such thinning and/or stratocumulus‐to‐cumulus transitions.

### 5.2 Differences in Cloud Optical Feedback

The cloud optical feedback states that an increase in CLWC under warming would increase cloud albedo, a cooling effect. CLWC may be expected to increase under global warming for three reasons. First, if one assumes that the amount of cloud water within a given cloud follows a moist adiabat, then one can show that CLWC always increases with temperature (Betts & Harshvardhan, 1987; Ceppi et al., 2017). Second, the increased humidity expected with warmer surface temperatures (due to the increased saturation mixing ratio) implies a greater availability of water vapor for condensation (Somerville, 1985). Third, for so‐called mixed phase clouds (clouds containing both liquid water and ice), increases in temperature would promote phase changes from ice to liquid, implying a greater proportion of cloud water (Ceppi et al., 2017). In all cases, the effect will be to increase the cloud optical depth (and hence albedo), implying that an increase in CLWC with warming is a potential *negative* feedback. Modern GCMs corroborate this, typically exhibiting a small, global net negative cloud optical feedback (Ceppi et al., 2017).

One simple way of measuring the potential strength of this feedback due to the first mechanism above was outlined in Somerville (1985) (see also Betts & Harshvardhan, 1987 and Charlock, 2012), which we will apply here. For clouds within the approximate temperature range −25 to 0 °C, both observational data and models show that CLWC increases with increased temperatures, roughly in line with what is predicted by the moist adiabatic approximation. By stratifying clouds according to temperature, we can compute the gradient *λ* of CLWC with respect to changes in temperature, by fitting a straight line to the data. The larger is *λ*, the more CLWC would be expected to increase per unit kelvin. Therefore, the magnitude of *λ* gives a first‐order estimate of how strong (i.e., how negative) the cloud optical feedback in the model is.

We estimated this metric for clouds in the FastSPHINX experiments for pressure levels 850, 700, and 500 hPa, which span the approximate range for which we find clouds in the right temperature range. The mean value of *λ* across the full deterministic ensemble at 700 hPa is 3.62·10^−6^ kg·m^−2^·K^−1^, with a standard error of 0.117·10^−6^ kg·m^−2^·K^−1^. The stochastic mean is 3.81·10^−6^ kg·m^−2^·K^−1^, with a standard error of 0.046·10^−6^ kg·m^−2^·K^−1^. The difference is statistically significant and represents an increase of around 5% with SPPT. An almost identical, statistically significant increase is found at the 500‐hPa level. However, at 850 hPa, there is no statistically significant change in the gradient across the two ensembles. This may again be due to the tapering of the stochastic perturbations near the surface, which imply a weakening of the scheme's impact at lower pressure levels.

As noted previously, the difference between the stochastic and deterministic ensemble mean CLWC stays roughly constant in time, despite the stochastic simulations warming less, consistent with the above calculations. While changes in CLWC cannot be easily untangled from changes in cloud cover, already seen to be different for the two sets of simulations, this suggests that SPPT has increased the negative cloud optical feedback.

Note that because CLWC was not available on levels in the SPHINX simulations, the computation of *λ* could not be carried out for these. We cannot therefore rule out that this change in *λ* may be a feature of the spin‐up phase only.

### 5.3 Nonlinearity of Climate Sensitivity

There is some evidence that there is a dependence of the amount of global warming (due to some fixed forcing) on the initial global mean temperature (e.g., Bloch‐Johnson et al., 2015; Caballero & Huber, 2013; Friedrich et al., 2016; Meraner et al., 2013). These studies suggest that the system's sensitivity may increase with increased temperatures, for example, due to an increase in the water vapor feedback (Meraner et al., 2013). Because the stochastic simulations start with a slightly cooler mean state, it is possible that the reduced GHG sensitivity may be due to this effect.

The exact extent of this dependence on the initial state is not well understood, and so the attribution of a 10% decrease due to a mean state ∼0.3 K cooler cannot be done rigorously. We will therefore simply demonstrate, given one example from the literature, that this hypothesis cannot be immediately discounted. Specifically, Friedrich et al. (2016) use reconstructed paleoclimatic data to estimate climate sensitivity starting from a glacial period or an interglacial period. In the cooler glacial period, they find a sensitivity to doubling CO_2_ of 1.78 K, while in the warmer interglacial period their estimate increases to 4.88 K. Under the assumption that climate sensitivity scales linearly with temperature, this implies an approximately 55% increase in sensitivity per Kelvin temperature increase. A 0.3‐K difference would, using these estimates, imply a difference in sensitivity of around 16%. Consequently, the decreased climate sensitivity with SPPT may be due at least partially to the altered mean state.

## 6 Discussion and Conclusions

We have demonstrated that the inclusion of a stochastic scheme in a coupled climate model can significantly change not only the model's mean surface temperature but also its GHG sensitivity. Concretely, in the EC‐Earth model considered here, the inclusion of the SPPT scheme led to a cooling of the model's mean surface temperature by ∼0.3 K, as well as decreasing the magnitude of global warming by around 10%. It is possible that the ultimate difference, after allowing the simulations to reach a new equilibrium, may be larger still. We linked the change in the model's historical climate to a rapid increase in cloud liquid water when turning on SPPT, which we speculated was due to the nonlinear impact of perturbations on condensation triggers. A similar increase had been noted also by Strømmen et al. (2019), using a different version of EC‐Earth, suggesting that this may be a robust impact of the scheme. The increased cloud liquid water amplifies the cloud albedo, resulting in a cooling, which is never fully compensated for by subsequent climate feedbacks. For the decrease in climate sensitivity, three possible mechanisms were suggested. First, the stochastic scheme appears to slow down the steep decline in low‐level cloud cover observed in the model as the surface temperature increases: This implies a reduction in the positive low cloud cover feedback in the stochastic model. Second, the negative cloud optical feedback appears to be slightly amplified, again implying reduced sensitivity. Finally, some role may be played by possible nonlinearity of climate sensitivity, for example, due to the nonlinearity of water vapor feedback observed in some studies.

These results reinforce a growing body of evidence showing that the inclusion of a stochastic scheme can, through nonlinear processes, shift a model to a notably different climate attractor. In particular, the choice of a stochastic scheme (including not having one at all) can significantly impact both a GCMs reproduction of historical data, as well as its projections of future climate change. However, some important outstanding questions remain. Most importantly, is the impact on the cloud cover feedback an effect of the changed mean state alone, or is there a more dynamic interaction of SPPT with the cloud thinning processes? In other words, would the models' mean state climate sensitivities be more similar if the stochastic model were tuned in a similar way to the deterministic model? If so, it is possible that the same changes (including, e.g., the increased cloud water) could have been achieved through parameter perturbations in the model: This would require further investigation to answer. Another outstanding question is the importance of conservation in stochastic schemes. The implementation of SPPT used in our experiments included a global “humidity fix” due to the scheme's nonconservation of water, and while changes to the mean state were shown to be independent of this, it is unclear if the fix is influencing changes to GHG sensitivity. It should be noted that alternative stochastic schemes have been constructed that are more conservative by construction; one example of such a scheme was introduced in Sanchez et al. (2015). Schemes based on parameter perturbations, such as that described in Leutbecher et al. (2017), are also conservative by virtue of only perturbing the underlying equations instead of the tendencies themselves. Other physically motivated schemes, such as those described in Plant and Craig (2008) and Rochetin et al. (2014), may also better conserve mass and energy. It is the goal of the authors to revisit these questions in the context of SPPT in future work. More generally, it is important to note that the impact of stochastic schemes other than SPPT may be very different in nature, including not influencing the sensitivity at all.

In light of our results, it would be of great interest to assess further the impact of SPPT on the key, small‐scale processes driving cloud feedbacks. This could potentially inform the development of new stochastic schemes. Given the ability of such schemes to improve other key aspects of models, this may, ultimately, lead to more accurate projections of global warming.
